# FoxM1 and β-catenin predicts aggressiveness in Middle Eastern ovarian cancer and their co-targeting impairs the growth of ovarian cancer cells

**DOI:** 10.18632/oncotarget.23338

**Published:** 2017-12-16

**Authors:** Poyil Pratheeshkumar, Sasidharan Padmaja Divya, Sandeep Kumar Parvathareddy, Norah M. Alhoshani, Ismail A. Al-Badawi, Asma Tulbah, Fouad Al-Dayel, Abdul K. Siraj, Khawla S. Al-Kuraya

**Affiliations:** ^1^ Human Cancer Genomic Research, King Faisal Specialist Hospital and Research Center, Riyadh, Saudi Arabia; ^2^ Department of Obstetrics and Gynecology, King Faisal Specialist Hospital and Research Centre, Riyadh, Saudi Arabia; ^3^ Department of Pathology, King Faisal Specialist Hospital and Research Centre, Riyadh, Saudi Arabia

**Keywords:** EOC, FoxM1, β-catenin, thiostrepton, FH535

## Abstract

Epithelial ovarian cancer (EOC) is a highly lethal disease with poor prognosis especially in advanced stage tumor. Emerging evidence has reported that aberrant upregulation of FoxM1 and β-catenin are closely associated with aggressiveness of human cancer. However, interplay between these factors in the aggressiveness of EOC is not fully illustrated. In this study, we show that FoxM1 is frequently increased in Middle Eastern EOC and associated with high proliferative index (*p* = 0.0007) and high grade tumor (*p* = 0.0024). Interestingly, FoxM1 is significantly associated with elevated nuclear β-catenin and the concomitant increase of FoxM1 and β-catenin is associated with advanced stage of EOC by immunohistochemical analysis of 261 samples of Saudi patients with EOC. Functional analysis showed that β-catenin is a direct transcriptional target of FoxM1 in EOC cell lines. FoxM1 inhibition either by specific inhibitor, thiostrepton or siRNA suppressed β-catenin expression, whereas overexpression of FoxM1 increased nuclear β-catenin expression. We identified two FoxM1 binding sites in the β-catenin promoter that specifically bound to FoxM1 protein. Down-regulation of FoxM1 using thiostrepton induced apoptosis and inhibited cell migration/invasion in EOC cells. Moreover, co-inhibition of FoxM1 by thiostrepton and β-catenin by FH535 significantly and synergistically inhibited EOC cell growth *in vitro* and *in vivo*. Collectively, our findings confer that co-targeting FoxM1/β-catenin signaling cascade may be a promising molecular therapeutic choice in advanced EOC.

## INTRODUCTION

Ovarian cancer is the most lethal and the second most commonly diagnosed disease among all gynecological malignancies worldwide [1–3]. The high mortality rate of ovarian cancer is due to its poor prognosis and majority of the cases are diagnosed with advanced stage disease [4]. Ovarian cancer is a heterogeneous tumor with a wide range of clinical presentations, cytological features and genetic alterations [5, 6]. Ovarian high-grade cancers are characterized by high-grade nuclei, poor differentiation, a high mitotic index, less responsive to chemotherapy, more aggressiveness as well as low survival rate [6–8]. The relatively worse pathogenesis and clinicopathologic features of high-grade ovarian cancers cause poor clinical management of this type of disease. Therefore, understanding the underlying molecular mechanisms may assist in developing better curative therapy in aggressive ovarian cancers.

Forkhead box M1 (FoxM1) is a member of the evolutionary conserved Forkhead box transcription factor family, with a conserved winged-helix DNA-binding domain [9]. Emerging evidence has shown that aberrant upregulation of FoxM1 is seen frequently in various human cancers [10–13]. FoxM1 has been shown to play a role in tumor invasion, migration, and angiogenesis [14–16]. Given that FoxM1 acts as a critical master regulator of tumorigenesis and metastasis in human cancers, it is imperative to understand the molecular mechanism of FoxM1 involved in the transcriptional regulation of the diverse signaling pathways in each step of tumorigenesis. The identification of downstream regulators of FoxM1 might provide potentially reliable molecular therapeutic target for ovarian cancer.

The WNT/β-catenin Pathway has shown to contribute to ovarian cancer initiation, metastasis, chemo-resistance and recurrence [17–19]. The oncogenic potential of FoxM1 is mainly based on its ability to transcriptionally activate genes that are involved in different facets of cancer development [20]. Activation of TCF4/LEF-1 by binding to β-catenin induces the transcription of various target genes, including c-Myc, cyclin D1, VEGF, MMP2 and MMP9 [21–25]. Therefore, it is important to elucidate how aberration in components of the Wnt signaling pathway causes the activation of β-catenin/TCF4-mediated transcription in tumors, including EOC.

It has been reported that FoxM1 directly binds to β-catenin and increases β-catenin nuclear localization and transcriptional activation of WNT target genes and glioma tumorigenesis [24]. In addition to glioma, several other tumors have shown the upregulation of both FoxM1 and WNT/β-catenin including medulloblastoma, colon cancer and hepatocellular carcinoma [26–28]. In this study, we show using a large cohort of Middle Eastern ovarian cancer that co-expression of FoxM1 and β-catenin were significantly correlated with advanced stage. We also describe the regulatory mechanism of FoxM1/β-catenin and the inhibition of these signaling using the FoxM1 inhibitor, thiostrepton and β-catenin inhibitor, FH535 could synergistically abrogate the ovarian cancer growth, migration/invasion as well as *in vitro* and *in vivo* tumor growth. Our results emphasize the importance of FoxM1/β-catenin interaction in ovarian tumorigenesis and argue the importance of this pathway as a promising therapeutic target in high-grade ovarian cancer.

## RESULTS

### Evaluation of FoxM1 over-expression by IHC in EOC TMA

**Figure 1:**
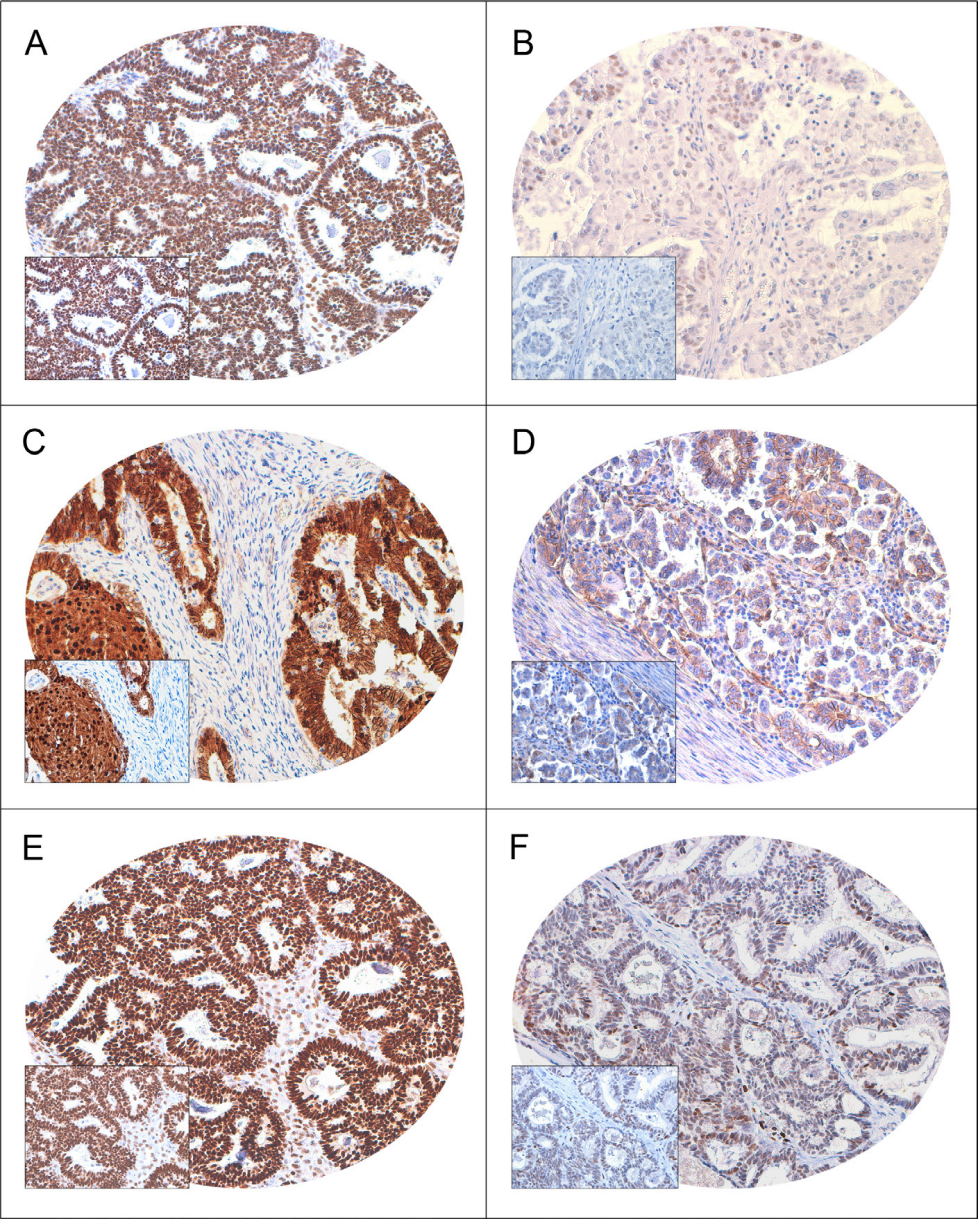
Immunohistochemical analysis of FoxM1, β-catenin and TCF4 expression in Epithelial Ovarian Cancer (EOC) TMA (*n* = 261) A EOC array spot showing overexpression of FoxM1 (**A**), β-catenin (**C**) and TCF4 (**E**). In contrast, another EOC tissue array spots showing low expression of FoxM1 (**B**), β-catenin (**D**) and TCF4 (**F**).

Immunohistochemical analysis of FoxM1 expression was interpretable in 261 EOC spots and the incidence of FoxM1 over-expression was found to be 60.5% (158/261). FoxM1 expression was seen predominantly in the nuclear compartment. FoxM1 over-expression was associated with adverse clinico-pathological parameters such as high grade serous carcinomas (*p* = 0.0221), poorly differentiated tumors (*p* = 0.0024) and high proliferative index (Ki-67, *p* = 0.0007) (Table 1). Of particular interest was the significant association between FoxM1 over-expression and elevated nuclear β-catenin expression (*p* = 0.0139). This concomitant increase of FoxM1 and β-catenin was associated with advanced stage (Stage III and IV, *p* = 0.0389) EOCs, thus providing a clue to the possible role of interplay between these two markers in promoting aggressiveness of EOCs (Supplementary Table 1). Significant association of FoxM1 over-expression was also noted with transcriptional factor TCF4 (*p* = 0.0066); markers of invasion and migration, MMP-9 (*p* = 0.0455) and u-PAR (0.0071), and cell cycle regulator, Cyclin D1 (*p* = 0.0094) (Figure 1, Table 1).

**Table 1:** Association of clinico-pathological characteristics with FoxM1 over-expression in patients with epithelial ovarian cancer

	Total		High Fox-M1		Low Fox-M1		*p* value
	*N*	%	*N*	%	*N*	%	
Total Number of Cases	261		158	60.5	103	39.5	
Age							
≤ 50 years	117	44.8	67	57.3	50	42.7	0.3300
>50 years	144	55.2	91	63.2	53	36.8	
Histopathology							
High grade Serous	138	52.9	97	70.3	41	29.7	0.0221
Low grade Serous	55	21.1	26	47.3	29	52.7	
Mucinous	27	10.3	12	44.4	15	55.6	
Endometriod	27	10.3	16	59.3	11	40.7	
Clear cell	6	2.3	3	50.0	3	50.0	
Undifferentiated	8	3.1	4	50.0	4	50.0	
FIGO Grade							
Well differentiated	50	19.2	23	46.0	27	54.0	0.0024
Moderately Differentiated	114	43.7	64	56.1	50	43.9	
Poorly Differentiated	97	37.1	71	73.2	26	26.8	
Tumour Stage							
Stage I	34	13.6	20	58.8	14	41.2	0.2397
Stage II	13	5.2	10	76.9	3	23.1	
Stage III	159	63.4	92	57.9	67	42.1	
Stage IV	45	17.9	32	71.1	13	28.9	
Ki-67							
Above 50	135	53.1	96	71.1	39	28.9	0.0007
Below = 50	119	46.9	60	50.4	59	49.6	
β-catenin							
Above 0	41	16.3	32	78.0	9	22.0	0.0139
Below = 0	211	83.7	123	58.3	88	41.7	
TCF4							
Above 90	216	84.0	139	64.4	77	35.6	0.0066
Below = 90	41	16.0	17	41.5	24	58.5	
VEGF							
Above 140	47	18.4	31	66.0	16	34.0	0.5056
Below = 140	209	81.6	127	60.8	82	39.2	
MMP-2							
Above 80	204	80.9	129	63.2	75	36.8	0.0531
Below = 80	48	19.1	23	47.9	25	52.1	
MMP-9							
Above 80	190	81.2	122	64.2	68	36.8	0.0455
Below = 80	44	18.8	21	47.7	23	52.3	
Cyclin D1							
Above 15	51	19.7	39	76.5	12	23.5	0.0094
Below = 15	208	80.3	119	57.2	89	42.8	
u-PAR							
Above 50	189	75.3	125	66.1	64	33.9	0.0071
Below = 50	62	24.7	29	46.8	33	53.2	
PFS–Median (months)				20.3		18.4	0.3869

Contingency table analysis and χ2 tests were used to study the relationship between clinico-pathological variables and protein expression. Survival curves were generated using the Kaplan-Meier method, with significance evaluated using the Mantel-Cox log-rank test. The limit of significance for all analyses was defined as a *p*-value of 0.05.

We further analyzed the expression of FoxM1 in high grade serous carcinoma and low grade serous carcinoma. Our results showed that incidence of FoxM1 overexpression is higher in the high grade serous tumors than low grade serous tumors – 70.3% (97/138) vs 47.3% (26/55). We also observed that FoxM1 overexpression is associated with high proliferative index (Ki67, *p* = 0.0072) in high grade serous carcinoma. Interestingly, only in high grade serous carcinoma FoxM1 overexpression showed significant association with elevated nuclear β-catenin expression (*p* = 0.0089) (Supplementary Tables 2 and 3).

### FoxM1 interact with β-catenin *in vitro* and *in vivo* in EOC

**Figure 2:**
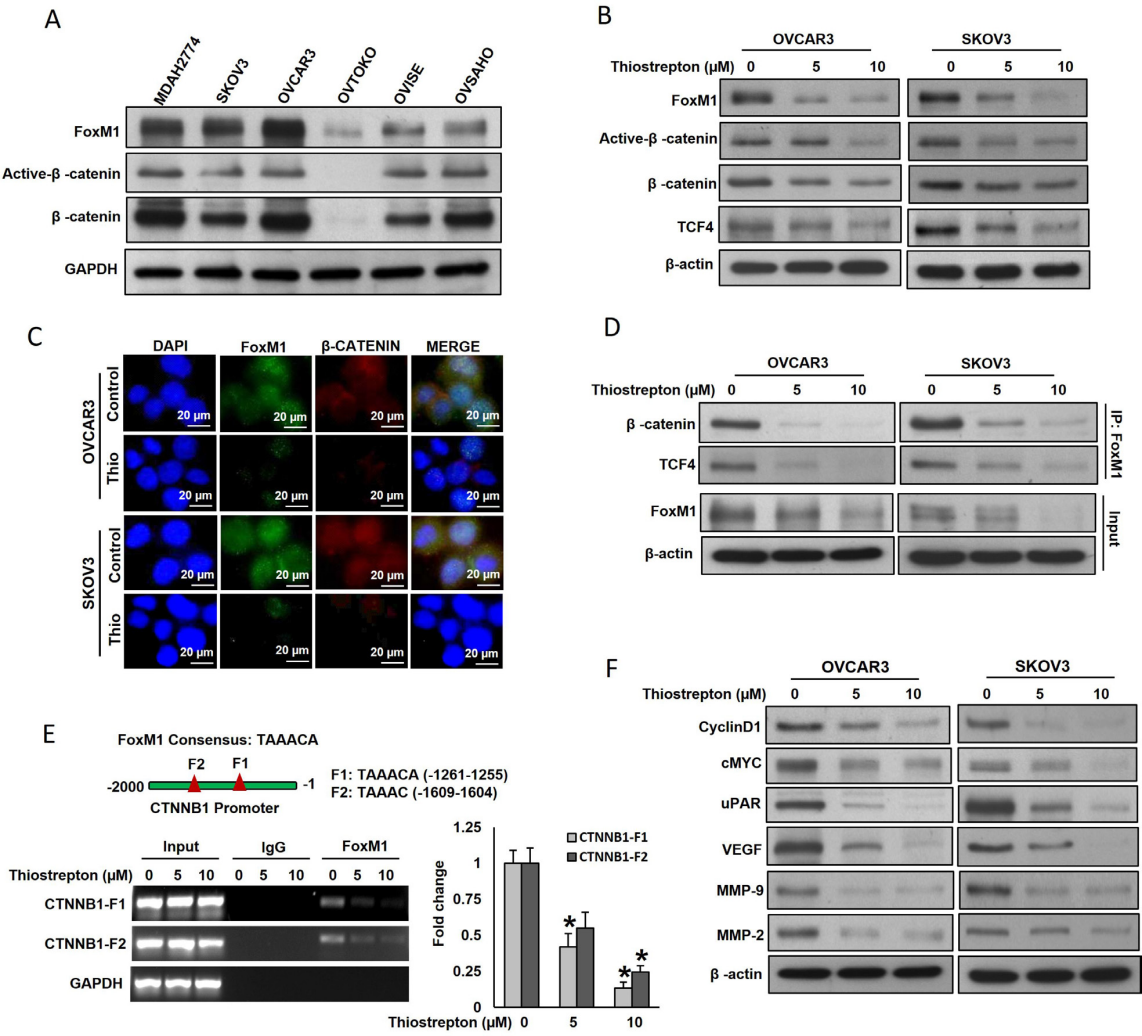
FoxM1 interact with β-catenin *in vitro* (**A**) Basal expression of FoxM1, β-catenin and TCF4 in EOC cells. Proteins were isolated from six EOC cell lines and immunoblotted with antibodies against FoxM1, Active-β-catenin, β-catenin, and GAPDH. (**B**) Thiostrepton inhibits β-catenin and TCF4 expression in EOC cells. EOC cells were incubated with indicated doses of thiostrepton for 48 hours. Proteins were isolated and immunoblotted with antibodies against FoxM1, active-β-catenin, β-catenin, TCF4 and β-actin. (**C**) Localization of FoxM1 and β-catenin in EOC cells using immunofluorescence analysis. (**D**) FoxM1 interact with β-catenin. Cell lysates extracted from with and without thiostrepton treated EOC cells were immunoprecipitated with FoxM1 antibody. Interaction of endogenous FoxM1 and β-catenin was detected by immunoblotting. (**E**) FoxM1 binding to β-catenin promoter. For the ChIP analysis, the FoxM1 binding regions on β-catenin promoter were identified. OVCAR3 cells were treated with and without indicated doses of thiostrepton. After 48 hours, cells were fixed with formaldehyde and cross-linked. The chromatin was sheared and immunoprecipitated with anti-FoxM1 antibody or control mouse IgG. The FoxM1 binding to the β-catenin promoter was analyzed using specific primers. ^*^p < 0.05, statistically significant difference from control cells, (*n* = 2). (**F**) Thiostrepton inhibits β-catenin/TCF4 downstream targets in EOC cells. EOC cells were incubated with indicated doses of thiostrepton for 48 hours. Proteins were isolated and immunoblotted with antibodies against Cyclin D1, cMYC, uPAR, VEGF, MMP-9, MMP-2 and β-actin.

Our clinical data showed that FoxM1 was significantly associated with elevated nuclear β-catenin. To study the FoxM1 and β-catenin interaction *in vitro*, we first detected the basal expression of FoxM1 and β-catenin in a panel of six cell lines by immuno-blotting. As shown in Figure 2A, we identified concomitant expression of FoxM1 and β-catenin in five EOC cell lines (MDAH2774, SKOV3, OVCAR3, OVISE and OVSAHO), whereas cells with low or negligible expression of FoxM1 (OVTOKO) showed low expression of β-catenin. Next, we inhibited FoxM1 using a specific FoxM1 inhibitor, thiostrepton and analyzed the expression of β-catenin and TCF4 in EOC cells. Figure 2B shows that inhibition of FoxM1 markedly down-regulated the expression of active-β-catenin and TCF4 in EOC cells in a dose-dependent manner. In order to determine the localization of FoxM1 and β-catenin, immunofluorescence staining in the EOC cells was employed (Figure 2C). The results show that untreated EOC cells exhibited a high expression of FoxM1 and β-catenin in nuclear region, while treatment of thiostrepton markedly decreased FoxM1 and β-catenin expression in the same region (Figure 2C). TCF4 expression was also abundant in the nucleus of FoxM1 expressing EOC cells, while inhibition of FoxM1 using thiostrepton down-regulates TCF4 expression (Supplementary Figure 1A). Next, physical interaction of FoxM1 with β-catenin was examined by immunoprecipitation (IP) analysis. The result shows that FoxM1 binds to β-catenin and TCF4 and was dramatically reduced after thiostrepton treatment in EOC cells (Figure 2D). To determine whether FoxM1 transcriptionally activates β-catenin in our model, we performed ChIP analysis. Nucleotide sequence analysis of the 2-kb β-catenin promoter revealed the presence of two FoxM1 consensus sequences in the forward strand (Figure 2E). ChIP analysis demonstrated that FoxM1 binds to β-catenin promoters at both sites, F1 (-1261-1255) and F2 (-1609-1604) in OVCAR3 cells. Interestingly, the degree of FoxM1 binding to β-catenin promoter at both sites was decreased after thiostrepton treatment in a dose dependent manner (Figure 2E).

It has been reported that uPAR, c-Myc, cyclinD1, VEGF and MMPs are the target genes of β-catenin/TCF4 dependent transcription [29–31]; and these genes have been implicated in different cellular processes including proliferation, survival, migration, invasion and angiogenesis [32]. As shown in Figure 2F, thiostrepton treatment decreased the CyclinD1, c-Myc, uPAR, VEGF, MMP-9 and MMP-2 expressions in EOC cells in a dose-dependent manner. Reports indicated that TCF4 binds to uPAR and cMYC promoters [29, 33]. To confirm this in our model system, we performed ChIP analysis using a TCF4 antibody and primers that specifically amplify the β-catenin/TCF4 binding site on the promoters of uPAR (−308 to −302) and c-Myc (−452 to −446). As shown in Supplementary Figure 1B-1C, TCF4 binds to the uPAR and c-Myc promoters in OVCAR3 cells and the degree of binding was decreased after thiostrepton treatment in a dose dependent manner.

**Figure 3:**
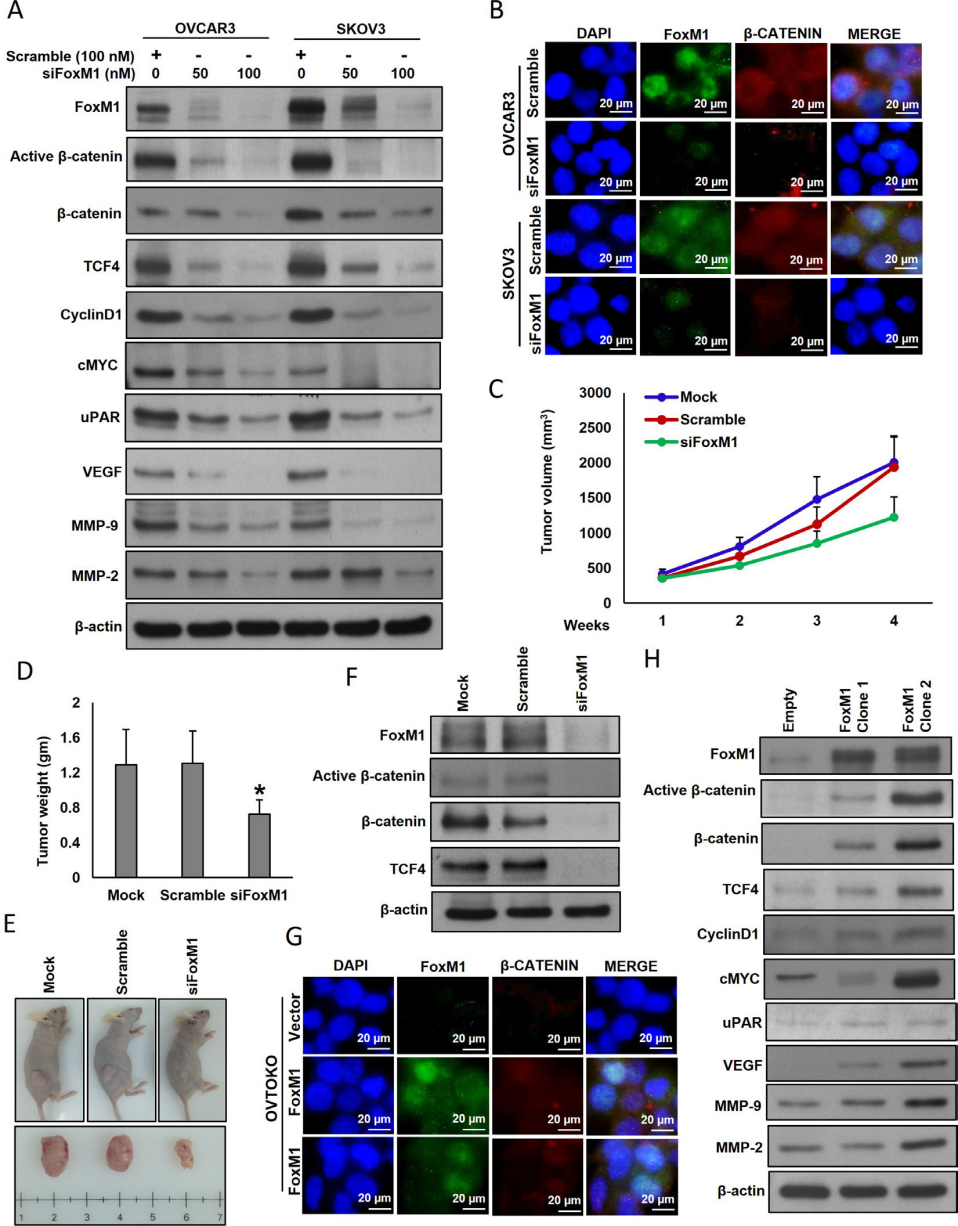
Effect of FoxM1 silencing and forced expression in EOC cell lines Silencing of FoxM1 down regulates the expression of β-catenin and their downstream targets as well as inhibits tumorigenesis in nude mice. (**A**) Silencing of FoxM1 inhibits β-catenin and associated downstream targets. EOC cells were transfected with scrambled siRNA and FoxM1 siRNA (50 and 100 nM). After 48 hours, cells were lysed and proteins were immunoblotted with antibodies against FoxM1, active-β-catenin, β-catenin, TCF4, Cyclin D1, cMYC, uPAR, VEGF, MMP-9, MMP-2 and β-actin. (**B**) Representative images of fluorescence immunostaining for FoxM1 and β-catenin in EOC cells after post-transfection with FoxM1 siRNA. (**C**–**E**) Silencing of FoxM1 inhibits tumorigenesis in nude mice. OVCAR3 cells were transfected with scrambled siRNA and FoxM1 siRNA (100 nM). Cells (2 × 10^6^ cells) were injected into the flanks of 6-week old nude mice (*n* = 5). Solid tumors in the FoxM1 silenced OVCAR3 cells injected mice were significantly smaller than those mice injected with OVCAR3 alone or OVCAR3 transfected with scramble siRNA. Silencing of FoxM1 significantly reduced (C) tumor volume, and (D) tumor weight in the mice injected with FoxM1 silenced SKOV3 cells. (E) Representative tumor images of xenografts. (**F**) Tissue lysates were immuno-blotted with FoxM1, active-β-catenin, β-catenin, TCF4, and β-actin antibodies. (**G**–**H**) Forced expression of FoxM1 induces β-catenin and associated downstream targets. (G) Representative images of fluorescence immunostaining for FoxM1 and β-catenin in OVTOKO cells after post-transfection with FoxM1 plasmid. (H) OVTOKO cells were transfected with either empty vector or FoxM1 plasmid for 48 hours. Proteins were isolated and were immuno-blotted with antibodies against FoxM1, active-β-catenin, β-catenin, TCF4, Cyclin D1, cMYC, uPAR, VEGF, MMP-9, MMP-2 and β-actin.

To confirm these above findings, we silenced FoxM1 with specific siRNA and assessed the protein expression of FoxM1 and their downstream targets in EOC cells. As shown in Figure 3A, silencing of FoxM1 exhibited similar results of inhibiting FoxM1 with thiostrepton. Silencing of FoxM1 decreased active-β-catenin and TCF4 expression with an accompanying downregulation of CyclinD1, cMYC, uPAR, VEGF, MMP-9 and MMP-2 in EOC cells. These results were further confirmed by immunofluorescence analysis (Figure 3B). In addition, silencing of FoxM1 markedly reduced tumorigenesis in nude mice (Figure 3C–3E). Furthermore, tumors developed from FoxM1 knockdown cells showed decreased expressions of active-β-catenin and TCF4 compared to those tumors developed from non-transfected or scramble control cells (Figure 3F). Conversely, forced expression of FoxM1 in OVTOKO cells (FoxM1 low expressing cells) increased the accumulation of nuclear β-catenin (Figure 3G–3H) expression with an accompanied increase of TCF4, CyclinD1, cMYC, uPAR, VEGF, MMP-9 and MMP-2 expressions (Figure 3H). Next, we inhibited FoxM1 using thiostrepton and analyzed the expression of β-catenin and TCF4 in FoxM1 overexpressing OVTOKO clones. As shown in Supplementary Figure 2, treatment of thiostrepton dramatically down-regulated the expression of FoxM1, active-β-catenin and TCF4 in FoxM1 overexpressing clones.

### Down-regulation of FoxM1 using thiostrepton inhibited cell viability and induced apoptosis in EOC cells

**Figure 4:**
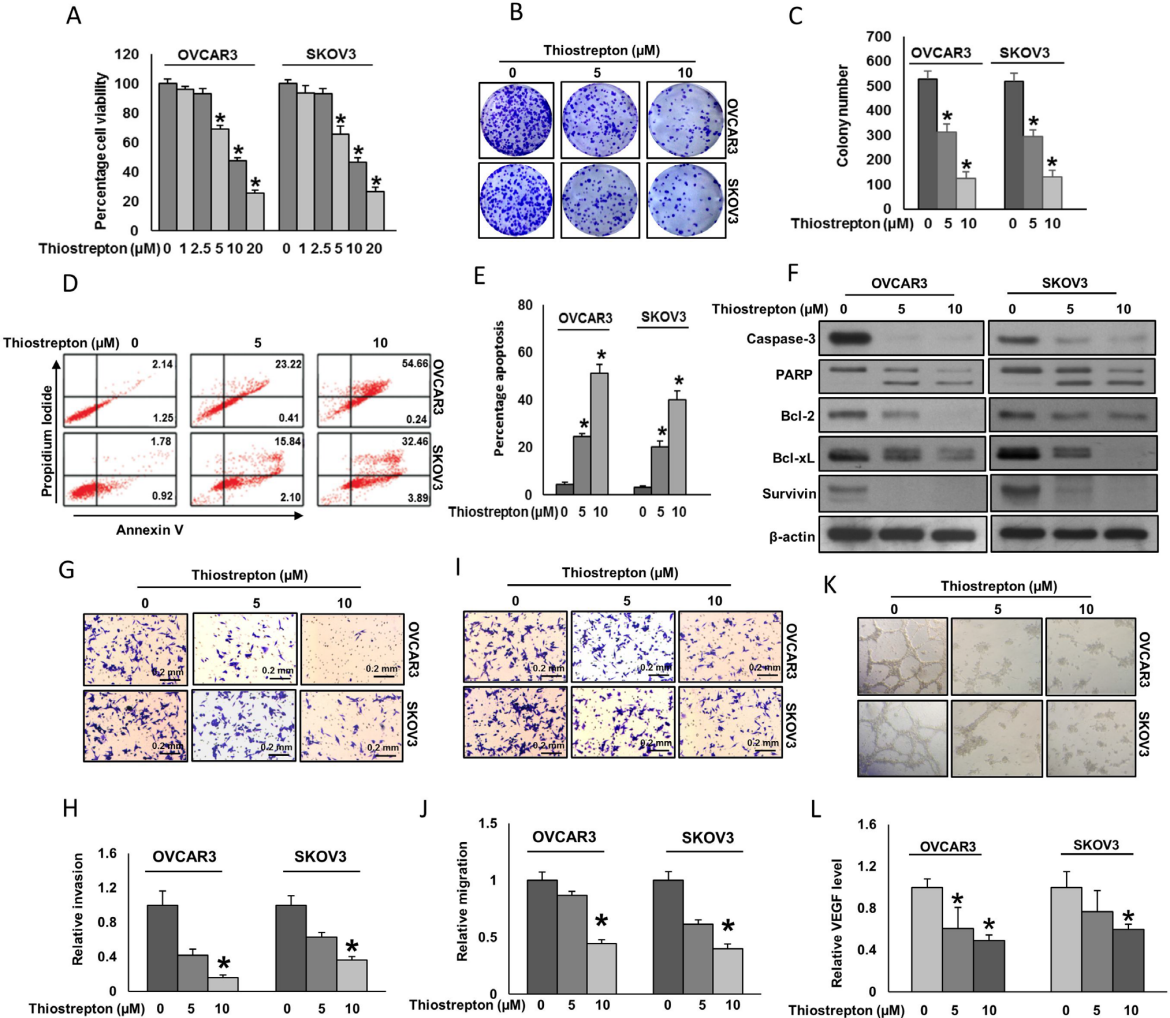
Effect of thiostrepton on cell viability, apoptosis, invasion, migration and angiogenesis (**A**) Thiostrepton inhibits cell viability. EOC cells were incubated with indicated doses of thiostrepton for 48 hours (*n* = 3). Cell viability was performed using MTT. (**B**–**C**) Thiostrepton inhibits clonogenicity. EOC Cells (8×10^2^) after thiostrepton treatment were seeded into each of three dishes (60 mm diameter), and grown for an additional 10 days, then stained with crystal violet and colonies were counted. (**D**–**E**) Thiostrepton induces apoptosis in EOC cell lines. EOC cells were treated with indicated doses of thiostrepton for 48 hours and cells were stained with flourescein-conjugated annexin-V and propidium iodide (PI) and analyzed by flow cytometry. Data presented in the bar graphs are the mean ± SD of three independent experiments. ^*^Indicates a statistically significant difference compared with control with *p* < 0.05. (**F**) Thiostrepton treatment causes activation of caspases and down-regulates the expression of anti-apoptotic proteins in EOC cells. EOC cells were treated with indicated doses of thiostrepton for 48 hours. After cell lysis, equal amounts of proteins were separated by SDS-PAGE, transferred to immobilon membrane, and immuno-blotted with antibodies against casapse-3, PARP, Bcl-2, Bcl-xL, survivin and β-actin as indicated. (**G**–**H**) FoxM1 inhibition causes reduction in the invasion capacity of EOC cells. EOC cells were pre-treated with universal caspase inhibitor, z-VAD/fmk (80 µM) for 3 hours and subsequently treated with indicated doses thiostrepton and seeded into the upper compartment of invasion chambers. The bottom chambers were filled with RPMI media. After 24 h incubation, invaded cells were fixed, stained and quantified (*n* = 2). (**I**–**J**) FoxM1 inhibition causes reduction in the migration capacity of EOC Cells. EOC cells were pre-treated with universal caspase inhibitor, z-VAD/fmk (80 µM) for 3 hours and subsequently treated with indicated doses thiostrepton and seeded into the upper compartment of migration chambers. The bottom chambers were filled with RPMI media. After 24 h incubation, migrated cells were fixed, stained and quantified (*n* = 2). (**K**) FoxM1 inhibition causes inhibition of HUVECs tube formation. HUVECs grown on matrigel were treated with conditioned media from thiostrepton treated and untreated EOCs for 24 h, cells were fixed, and tubular structures were photographed. (**L**) Thiostrepton inhibits VEGF secretion in EOC cells. EOC cells were treated with indicated doses of thiostrepton for 48 hours, and secreted VEGF level in the media was estimated by VEGF ELISA kit (Thermo Fisher Scientific) according to the manufacturers’ recommendations (*n* = 3). ^*^Indicates a statistically significant difference compared with control with *p* < 0.05.

We sought to determine whether down regulation of FoxM1 with thiostrepton, leads to inhibition of cell proliferation in EOC cells. EOC cell lines, OVCAR3 and SKOV3 were treated with different doses of thiostrepton for 48 hours and cell viability was assayed using MTT assay. Figure 4A shows a dose dependent and significant (*p* < 0.05) inhibition of cell viability in both cell lines. Similarly, thiostrepton treatment also significantly (*p* < 0.05) decreased the proliferation of EOC cells as confirmed by clonogenic assay (Figure 4B–4C). Next, to determine whether thiostrepton induced inhibition of cell viability was due to apoptosis, we treated EOC cells with increasing doses of thiostrepton for 48 hours and analyzed the cells for apoptosis after dual staining with annexin V/PI by flow cytometry. There was a dose dependent and significant induction of apoptosis in both EOC cell lines tested (Figure 4D–4E). As shown in Figure 4F, thiostrepton treatment induced activation and cleavage of caspase-3 and PARP in both cell lines. Furthermore, thiostrepton treatment also caused down-regulation of anti-apoptotic proteins, Bcl-2, Bcl-xL and survivin that play an important role in inhibition of apoptosis (Figure 4F). We found evidence that Bax protein underwent conformational changes at 16 hours in EOC cell lines after thiostrepton treatment at different time course (Supplementary Figure 3A). We then tested the effect of thiostrepton on the mitochondrial membrane potential and release of cytochrome c in these cells. As shown in Supplementary Figure 3B, treatment of cells with thiostrepton resulted in loss of mitochondrial membrane potential in EOC cells as measured by JC1 stained green fluorescence depicting apoptotic cells. We next studied the release of cytochrome c from mitochondria into cytosol. As shown in Supplementary Figure 3C, higher level of cytochrome c was measured in cytosolic and lower levels in mitochondrial fraction in both cell lines after thiostrepton treatment. Furthermore, we also pre-treated EOC cells with a universal caspase-inhibitor, zVAD-fmk for three hours followed by treatment with thiostrepton for 48 hours. The pre-treatment of zVAD-fmk significantly (*p* < 0.05) inhibited apoptosis induced by thiostrepton (Supplementary Figure 3D–3E). This data confirmed that thiostrepton-induced apoptosis is caspase dependent.

### Down-regulation of FoxM1 using thiostrepton inhibited invasion, migration and angiogenesis

FoxM1 has been shown to play a role in cell invasion, migration and angiogenesis via modulation of VEGF, uPAR, MMP-2 and MMP-9 [14], therefore, we next sought to investigate the effect of thiostrepton on cell invasion, migration and angiogenesis in EOC cells. As shown in Figure 2F, treatment of thiostrepton markedly decreased VEGF, uPAR, MMP-2 and MMP-9 expressions in EOC cells. To investigate whether down regulation of FoxM1 plays a role in inhibiting invasion and migration, EOC cell lines were treated with different doses of thiostrepton in the presence of universal caspase inhibitor (zVAD-fmk). Interestingly, inhibition of FoxM1 by thiostrepton significantly decreased invasion (Figure 4G–4H) and migration (Figure 4I–4J) of EOC cells. Treatment of zVAD-fmk confirms that inhibition of invasion and migration by thiostrepton was not through inducing apoptosis. We also studied the effect of FoxM1 inhibition in EOC cells on angiogenesis by tube formation assay. Tube formation of endothelial cells is one of the key steps of angiogenesis [34]. Incubation of HUVECs on matrigel with conditioned media (source of VEGF) of EOC cells resulted in the formation of elongated and tube like structures which were effectively reduced by conditioned media from thiostrepton treated EOC cells (Figure 4K). To further confirm these observations, we determined the secreted levels of VEGF by ELISA experiment in thiostrepton treated EOC cells. Decreased levels of VEGF was observed in a dose dependent manner in thiostrepton treated EOC cells (Figure 4L). Together, these findings suggest that thiostrepton can significantly repress FoxM1 expression and reduce EOC cell invasion, migration and angiogenesis by down-regulating FoxM1 target gene expression such as VEGF, uPAR, MMP-2 and MMP-9.

### Thiostrepton and FH535 synergistically inhibited EOC cell growth *in vitro* and *in vivo*

**Figure 5:**
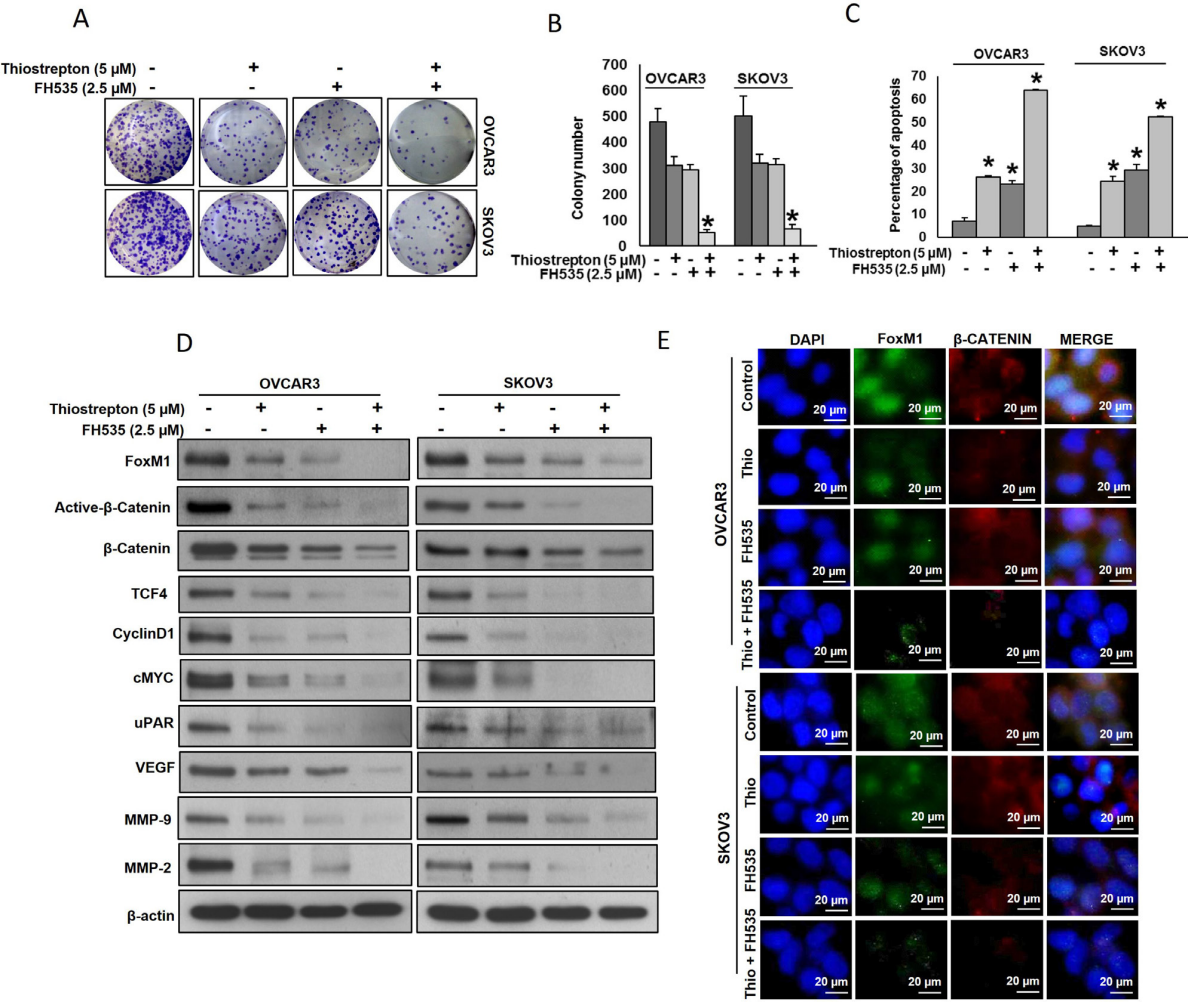
Thiostrepton and FH535 synergistically inhibits cell proliferation and induce apoptosis in EOC cell lines (**A**–**B**) Thiostrepton and FH535 synergistically inhibits clonogenicity. EOC Cells (8 × 10^2^) after thiostrepton and FH535 treatments were seeded into each of three dishes (60 mm diameter), and grown for an additional 10 days, then stained with crystal violet and colonies were counted. (**C**) Thiostrepton and FH535 synergistically induce apoptosis. EOC cells were treated with indicated doses of thiostrepton and FH535 either alone or in combination for 48 hours and cells were stained with flourescein-conjugated annexin-V and propidium iodide (PI) and analyzed by flow cytometry. Data presented in the bar graphs are the mean ± SD of three independent experiments. ^*^Indicates a statistically significant difference compared with control with *p* < 0.05. (**D**) Thiostrepton and FH535 synergistically inhibits FoxM1 and associated downstream targets. EOC cells were treated with indicated doses of thiostrepton and FH535 either alone or in combination for 48 hours. Proteins were isolated and were immunoblotted with antibodies against FoxM1, active-β-catenin, β-catenin, TCF4, Cyclin D1, cMYC, uPAR, VEGF, MMP-9, MMP-2 and β-actin. (**E**) Thiostrepton and FH535 synergistically inhibits FoxM1 and β-catenin expression as represented by fluorescence immunostaining in EOC cells.

Our clinical and *in vitro* data showed a strong association between FoxM1 and β-catenin protein expression in EOC, we assumed that targeting of FoxM1 and β-catenin expression together can lead to efficient cytotoxic effects in EOC cells. Therefore, we wanted to investigate whether co-treatment of thiostrepton and FH535 at sub-toxic doses, can potentiate anticancer effects in EOC cells. We have determined sub-optimal doses of thiostrepton and FH535 that can be used in combination to inhibit cell viability, clonogenicity and induce apoptosis. First we have treated sub-optimal doses of thiostrepton and FH535, either alone or in combination to EOC cells and analyzed the colony number. Combination of thiostrepton and FH535 significantly (*p* < 0.05) inhibited the clonogenicity of EOC cells as compared to treatment alone (Figure 5A–5B). As shown in Figure 5C, co-treatment of thiostrepton and FH535 also significantly (*p* < 0.05) potentiated apoptosis in EOC cells. Next we investigated whether co-treatment with sub-optimal doses of thiostrepton and FH535 could inhibit expression of FoxM1, β-catenin and their downstream targets by immuno-blotting. As shown in Figure 5D, combination treatment of thiostrepton and FH535 successfully down-regulated expression of FoxM1, active-β-catenin, β-catenin, TCF4, CyclinD1, cMYC, uPAR, VEGF, MMP-9 and MMP-2 in EOC cell lines. Similar results were observed by immunofluorescence where thiostrepton and FH535 synergistically inhibited the expression of FoxM1 and β-catenin in EOC cells (Figure 5E). These data clearly indicate that co-treatment with thiostrepton and FH535 synergistically inhibits cell viability and potentiate apoptosis in EOC cells.

**Figure 6:**
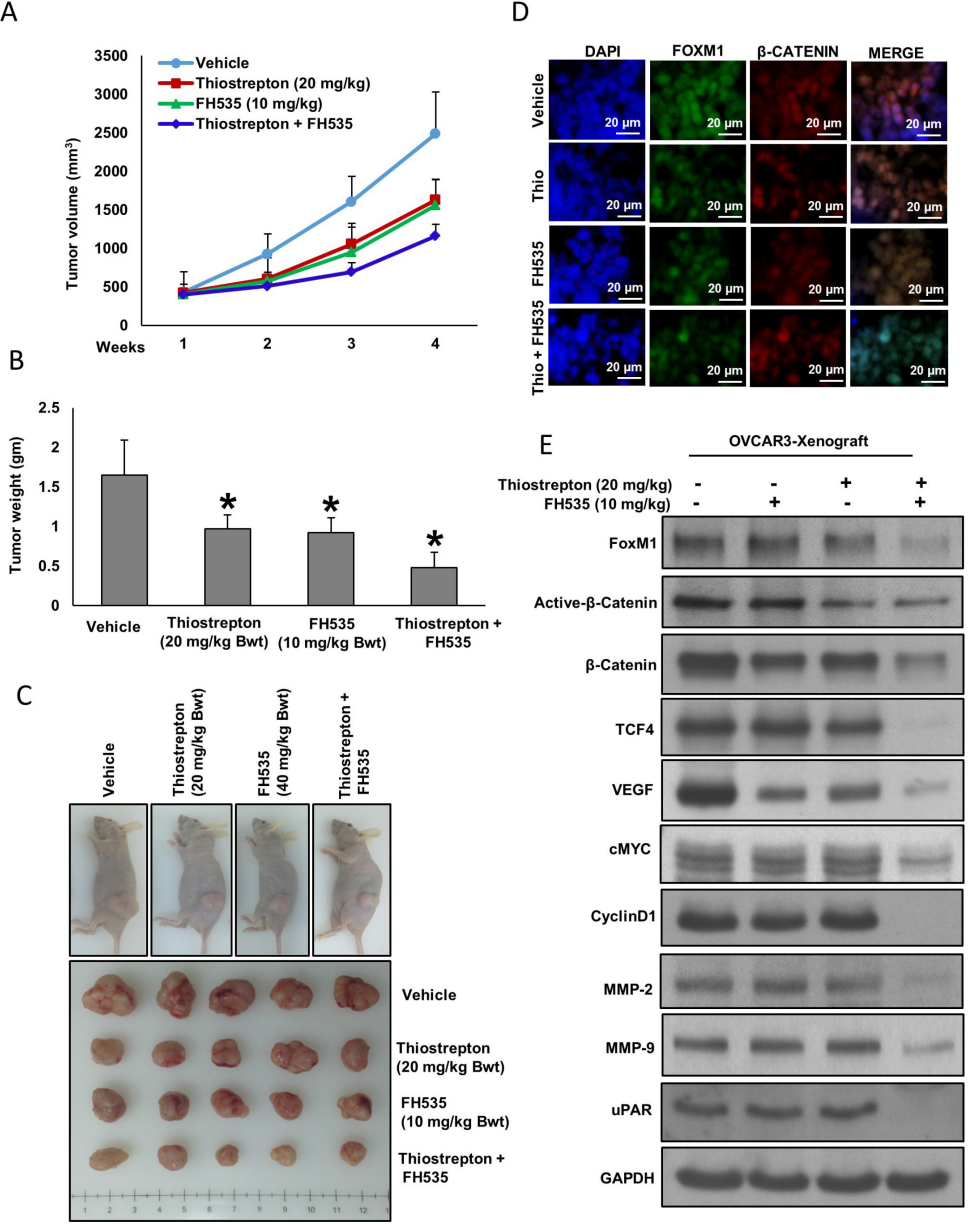
Thiostrepton and FH535 synergistically inhibits EOC tumor growth *in vivo* OVCAR3 cells were subcutaneously injected into the flanks of 6-week old nude mice (5 × 10^6^ cells per mouse). After tumors grew to about 100 mm^3^, mice were treated intraperitoneally with indicated dose of thiostrepton and FH535 either alone or in combination, twice a week for 30 days. (**A**) The volume of each tumor was measured every week. The average (*n* = 6) tumor volume in each group of mice was calculated. (**B**) After four weeks treatment, mice were sacrificed and mean tumor weight (± SD) was calculated in each group, ^*^p < 0.05. (**C**) Representative tumor images of each group of mice. (**D**) Fluorescence immunostaining for FoxM1 and β-catenin in xenograft tumors. (**E**) Tissue lysates from tumors were immuno-blotted with antibodies against FoxM1, active-β-catenin, β-catenin, TCF4, Cyclin D1, cMYC, uPAR, VEGF, MMP-9, MMP-2 and GAPDH.

The synergistic inhibition of FoxM1 and β-catenin significantly suppressed EOC cell growth *in vitro.* We further determined whether co-treatment of thiostrepton with FH535 suppress EOC cell line generated xenograft tumor growth in nude mice. For xenograft study, OVCAR3 cells were injected (5 × 10^6^ cells per mouse) into the flanks of 6-week-old female nude mice. After the tumors had developed (about 100 mm^3^), the mice were injected (i.p) with thiostrepton (20 mg/kg) and FH535 (10 mg/kg) either alone or in combination, twice a week for 30 days. DMSO (0.1%, i.p) was served as vehicle control. We found that co-treatment of thiostrepton and FH535 significantly suppressed tumor volume (Figure 6A) and tumor weight (Figure 6B) but had no effect on the body weight of mice (data not shown). Images of tumor showed that co-treatment of thiostrepton and FH535 caused shrinkage of tumor size (Figure 6C). Further, we analyzed the expression of FoxM1 and β-catenin in xenograft tumors by immunofluorescence and immuno-blotting. We observed both FoxM1 and β-catenin proteins accumulated in the nucleus of vehicle treated tumors (Figure 6D). Interestingly, co-treatment with thiostrepton and FH535 markedly suppressed the expression of FoxM1 and β-catenin (Figure 6D–6E) and their downstream target proteins in tumors (Figure 6E). Our data clearly indicates that co-treatment with thiostrepton and FH535 augmented antitumor effects in OVCAR3 cell xenografts in nude mice.

## DISCUSSION

In this study, we present that FoxM1 over-expression is a common alteration in Saudi EOC. FoxM1 is significantly associated with high proliferation and high grade EOC. Interestingly, FoxM1 is significantly associated with high expression of β-catenin. Importantly the concomitant increase of FoxM1 and β-catenin is associated with late stage (stage III and IV) EOC. In order to gain insight into biological plausibility of FoxM1/β-catenin overexpression in the pathogenesis of EOC, which would be suggested by our findings above, we first set out to test the potential role of FoxM1 in relevant EOC cell lines. To determine the role of FoxM1 expression *in vitro*, we assessed expression of FoxM1 in a panel of six EOC cell lines. On the basis of FoxM1 expression, we identified two EOC cell lines (OVCAR3 and SKOV3) that had over expression of FoxM1 as well as OVTOKO that had negligible expression. We functionally investigated the consequence of FoxM1 down-regulation in cell proliferation, apoptosis and angiogenesis as well as on invasion and migration. In our study, we found down-regulation of FoxM1 by thiostrepton elicited dramatic effect on cell viability and colony formation as confirmed by clonogenic assay. Down-regulation of FoxM1 by thiostrepton also induced apoptosis in OVCAR3 and SKOV3 cell line as confirmed by flow cytometry. We also tested the effect of the down regulation of FoxM1 by thiostrepton on the migration and invasion of EOC cells and tube formation (angiogenesis) of HUVECs. We found that the down-regulation of FoxM1 inhibited migration and invasion of EOC and reduced the tube formation of HUVECs.

Our clinical data showed FoxM1 expression and nuclear β-catenin are concomitantly increased in EOC samples and their upregulated expression are significantly associated with advance stage tumor, prompted us to study the biological relevance of this interaction. There are reports showing the biological interaction between FoxM1 and β-catenin in glioma [24], medulloblastoma [26, 35, 36] and colon cancer [37]. To determine this interaction, we first screened six EOC cell lines for the expression of FoxM1 and β-catenin by western blot. Interestingly, cell lines that displayed high level of FoxM1 showed activation of β-catenin whereas cell lines (OVTOKO) that had negligible expression of FoxM1 showed no activation of β-catenin. This concomitant expression of FoxM1 and β-catenin is similar to our findings in the clinical samples. Furthermore, we showed that down-regulation of FoxM1 by siRNA and thiostrepton resulted in decreased β-catenin transcriptional activity of Wnt target gene expression both *in vitro* and *in vivo*. We also confirmed the interaction between β-catenin and FoxM1 proteins in the nucleus by immunoprecipitation. This interaction was inhibited by down regulation of FoxM1 by thiostrepton in a dose dependent manner.

Importantly, we showed that FoxM1 binds to β-catenin promoter in the nucleus of EOC using ChIP analysis and this binding was inhibited with thiostrepton. Given this interaction of FoxM1-β-catenin signaling, we hypothesize that co-targeting both FoxM1 and β-catenin will be a novel therapeutic approach for late stage EOC. For this purpose, we used thiostrepton, FoxM1 inhibitor and FH535, a classic inhibitor of β-catenin pathway either alone or in combination, and our results showed the effectiveness of this therapeutic approach in inhibiting proliferation, inducing apoptosis and repressing EOC cancer cell growth and metastasis both *in vitro* and in EOC xenografts. Combination therapy was significantly better than monotherapy inhibition of EOC tumor growth. Combination with thiostrepton and FH535 was found to be synergistic not only in inhibition of cell growth but also in inhibition of metastatic and angiogenesis markers like MMP2, MMP9 and VEGF.

Altogether, our study showed the role of FoxM1 alteration in Middle Eastern EOC. Our data highlights the interaction between FoxM1 and β-catenin signaling pathways and suggest that co-targeting both FoxM1 and β-catenin might be a novel therapeutic approach in treatment of patient with aggressive late stage EOC.

## MATERIALS AND METHODS

### Clinical samples and TMA construction

Two hundred and seventy-six patients with EOC diagnosed between 1989 and 2015 were selected from King Faisal Specialist Hospital and Research Center. All samples were analyzed in a tissue microarray format. Tissue microarray construction was performed as described earlier [38]. Briefly, tissue cylinders with a diameter of 0.6 mm were punched from representative tumor regions of each donor tissue block and brought into recipient paraffin block using a modified semiautomatic robotic precision instrument (Beecher Instruments, Woodland, WI). Two 0.6-mm cores of EOC were arrayed from each case. Clinical and histological data were available for all these patients and are summarized in Supplementary Table 4. The patients were diagnosed histologically and received follow-up care in the Departments of Obstetrics and Gynecology and Oncology at King Faisal Specialist Hospital and Research Centre. Department of Obstetrics and Gynecology, King Faisal Specialist Hospital and Research Centre provided long-term follow-up data for these patients. The median follow-up time was 11.0 months (range, 2–199 months). Progression-free survival was computed from date of surgery for patients who underwent primary cytoreduction and from date of diagnosis by biopsy or cytology in those who underwent primary neoadjuvant chemotherapy. The institutional review board of the King Faisal Specialist Hospital and Research Centre approved the study.

### Immunohistochemistry (IHC)

Tissue microarray slides were processed and stained manually. The immunohistochemistry (IHC) protocol was followed as mentioned before [39] and described in Supplementary Materials and Methods .

FoxM1 immunohistiochemical expression was seen predominantly in the nuclear compartment and nuclear expression was quantified by H score as described previously [40]. Briefly, each TMA spot was assigned an intensity score from 0–3 (I_0_, I_1–3_) and proportion of the tumor staining for that intensity was recorded as 5% increments from a range of 0–100 (P_0_, P_1–3_). A final H score (range 0–300) was obtained by adding the sum of scores obtained for each intensity (I) and proportion (P) of area stained (H score = I_1_XP_1_ + I_2_XP_2_ + I_3_XP_3_) as described previously [41]. EOCs were categorized into 2 groups using X-tile bioinformatics software: low FoxM1 expression (H score ≤75) and high FoxM1 expression (H score>75). X-tile plots were constructed for assessment of biomarker and optimization of cut off points based on outcome as has been described earlier [42, 43]. Similarly, X-tile was used to determine an optimal cut off for other antibodies.

### Statistical analysis

Contingency table analysis and χ^2^ tests were used to study the relationship between clinico-pathological variables and protein expression. Survival curves were generated using the Kaplan-Meier method, with significance evaluated using the Mantel-Cox log-rank test. The limit of significance for all analyses was defined as a *p*-value of 0.05; two-sided tests were used in all calculations. The JMP10.0 (SAS Institute, Inc., Cary, NC) software package was used for data analyses.

### Cell culture

EOC cell lines MDAH2774, SKOV3, OVCAR3, OVSAHO, OVTOKO and OVISE cells were purchased from ATCC (Manassas, VA). Following tests of these cell lines for immunological markers and cytogenetics, they were also fingerprinted and species was confirmed by IEF of AST, MDH and NP. The cell lines were cultured in RPMI 1640 supplemented with 10% (v/v) fetal bovine serum (ATCC), 100 units/mL penicillin, and 100 units/mL streptomycin (SIGMA) at 37°C in humidified atmosphere containing 5% CO_2_. All experiments were performed in RPMI 1640 (ATCC) containing 5% serum.

### Reagents and antibodies

FoxM1 inhibitor, thiostrepton was purchased from Tocris Cookson Inc (Ellisville, MO). β-Catenin inhibitor, FH535 was purchased from Sigma-Aldrich (St. Louis, MO, USA). Antibodies against β-catenin (CST-9562), cMYC (CST-5605), MMP-9 (CST-2270), MMP-2 (CST-4022), caspase-3 (CST-9665), Bcl-2 (CST-2876) and Bcl-xL (CST-2762), β-actin (CST-3700) were purchased from Cell Signaling Technology (Beverly MA). Anti–active-β-catenin (clone 8E7#05-665) antibody was purchased from EMD Millipore (Billerica, MA). FoxM1 (sc-502), TCF4 (sc-166699), CyclinD1 (sc-753), uPAR (sc-10815), VEGF (sc-57496), Survivin (sc-374616), PARP (sc-7150) and GAPDH (sc-25778) antibodies were purchased from Santa Cruz Biotechnology, Inc. (Santa Cruz, CA). Annexin V was purchased from Molecular Probes (Eugene, OR).

### Gene silencing using siRNA

FoxM1 siRNA, and scrambled control siRNA were purchased from Qiagen (Valencia, CA, USA). Cells were transfected using Lipofectamine 2000 (Invitrogen, Carlsbad, CA) for 6 hours following which the lipid and siRNA complex was removed and fresh growth medium containing 20% fetal bovine serum was added. After 48 hours of transfection cells were harvested and used for various experiments such as immunofluorescence analysis, immuno-blotting and xenograft assays.

### Plasmid and transfection

Plasmid DNA encoding human FoxM1 was purchased from Origene (Rockville, MD). Transfection was performed using Lipofectamine™2000 (Invitrogen, Carlsbad, CA) according to the manufacturer’s protocol. Briefly, EOC cells were seeded in 6-well culture plates; when approximately 50% confluent, cells were transfected with 4 μg plasmid. After 48 hours of transfection, cells were used for immunofluorescence analysis and immuno-blotting.

### Immunofluorescence analysis

EOC cells grown on coverslips in 6-well plates were fixed with ice-cold 100% methanol followed by permeabilization with 0.2% Triton X-100, blocked with 5% horse serum in PBS solution, and incubated with antibodies to FoxM1 (1:100), β-catenin (1:200), or TCF4 (1:100) in buffer A (1% BSA, 0.1% Triton X-100, 10% horse serum in PBS solution) for 1 h at 37 °C. Cells were then incubated with Alexa Fluor 488 goat anti-rabbit or Alexa Fluor 594 goat anti-mouse antibody and mounted using DAPI. The cells were visualized using Olympus BX63 fluorescence microscope.

### Chromatin immunoprecipitation (ChIP) assay

ChIP analysis was performed using a Pierce agarose ChIP Kit (Thermo Scientific, Rockford, IL). OVCAR3 cells were treated with and without indicated doses of thiostrepton. After 48 hours of treatment, cells were fixed with formaldehyde and cross-linked. The chromatin was sheared and immunoprecipitated with 2 μg of anti-FoxM1or control IgG antibody. DNA protein complexes were eluted from protein A/G agarose beads using a spin column and were reverse cross-linked by incubating with NaCl at 65°C. The intensity of FoxM1 binding to the β-catenin promoter was analyzed by thermal cycler using following primer sequences, FoxM1 binding to the β-catenin promoter sites, F1(-1261-1255): (F) TGCTGCATTAGAATGGGAAA and (R) TGTGGGGATTTTTCTTTGGA; F2(-1609-1604): (F) AATTGGAGGCTGCTTAATCG and(R) TTGTGGGGATTTTTCTTTGG.

### Animals and xenografts study

Six-week-old nude mice were obtained from Jackson Laboratories (Bar Harbor, ME) and maintained in a pathogen-free animal facility at least 1 week before use. All animal studies were done in accordance with institutional guidelines. For xenograft study, mice were inoculated subcutaneously into the flanks with 5 × 10^6^ OVCAR3 cells in 100 µl PBS. After tumors grew to about 100 mm^3^, mice were treated intraperitoneally with thiostrepton (20 mg/kg) and FH535 (10 mg/kg) either alone or in combination, twice a week for 30 days. The body weight and tumor volume of each mouse was monitored weekly. After 5 weeks treatment, mice were sacrificed and individual tumors were weighed, then snap-frozen in liquid nitrogen for storage.

## SUPPLEMENTARY MATERIALS FIGURES AND TABLES


