# Supplementary material for: FoxM1 and β-catenin predicts aggressiveness in Middle Eastern ovarian cancer and their co-targeting impairs the growth of ovarian cancer cells
Source: Oncotarget. 2017 Dec 16;9(3):3590–604. doi: 10.18632/oncotarget.23338 (PMC5790485; doi:10.18632/oncotarget.23338)
Supplement: Supplementary file 1 [file oncotarget-09-3590-s001.pdf]

FoxM1 and β-catenin predicts aggressiveness in Middle Eastern ovarian cancer and their co-targeting impairs the growth of ovarian cancer cells

SUPPLEMENTARY MATERIALS

p53 mutation status of EOC cell lines

EOC cell lines MDAH2774, SKOV3, OVCAR3, and OVSAHO were p53 mutant, whereas OVTOKO and OVISE cells were found to be p53 wild type.

Measurement of mitochondrial membrane potential

cells were treated with thiostrepton for 48 hours, washed twice with PBS, and suspended in mitochondrial incubation buffer. JC1 was added to a final concentration of 10 µmol/L and cells were incubated at 37°C in dark for 15 min. Cells were then washed twice with PBS and resuspended in 500 µL of mitochondrial incubation buffer and mitochondrial membrane potential (% of green and red aggregates) was determined by flow cytometry.

Assays for cytochrome c release

Cells were treated with thiostrepton as described in figure legends, harvested, and resuspended in hypotonic buffer. Cells were homogenized and cytosolic and mitochondrial fractions were isolated by differential centrifugation. Twenty to 25 µg of protein from cytosolic and mitochondrial fractions of each sample were analyzed by immunoblotting using an anti–cytochrome c antibody.

Progression free survival data

The patients were diagnosed histologically and received follow-up care in the Departments of Obstetrics and Gynecology and Oncology at King Faisal Specialist Hospital and Research Centre. Department of Obstetrics and Gynecology, King Faisal Specialist Hospital and Research Centre provided long-term follow-up data for these patients. The median follow-up time was 11.0 months (range, 2–199 months). Progression-free survival was computed from date of surgery for patients who underwent primary cytoreduction and from date of diagnosis by biopsy or cytology in those who underwent primary neoadjuvant chemotherapy. Because the majority of patients are lost to follow-up as their disease reaches its terminal stages, it was impossible to determine overall survival in this specific patient population.

Immunohistochemical staining

The streptavidin–biotin peroxidase technique with diaminobenzidine as chromogen was used. The primary antibodies were diluted in a 1% solution of bovine serum albumin in phosphate-buffered saline (PBS) and incubated overnight at room temperature. Primary antibodies used, their dilutions and cutoff levels for evaluation are listed in Supplementary Table 5. For antigen retrieval, Dako Target Retrieval Solution (pH 9.0; catalog number S2368) was used, and antigen retrieval was carried out in a pressure cooker. The Dako Envision Plus System kit was used as the secondary detection system with DAB as chromogen. All slides were counterstained with hematoxylin, dehydrated, cleared and mounted. Negative controls included replacement of the primary antibody with no reacting antibodies of the same species. Only fresh cut slides were stained simultaneously to minimize the influence of slide aging and maximize reproducibility of the experiment.

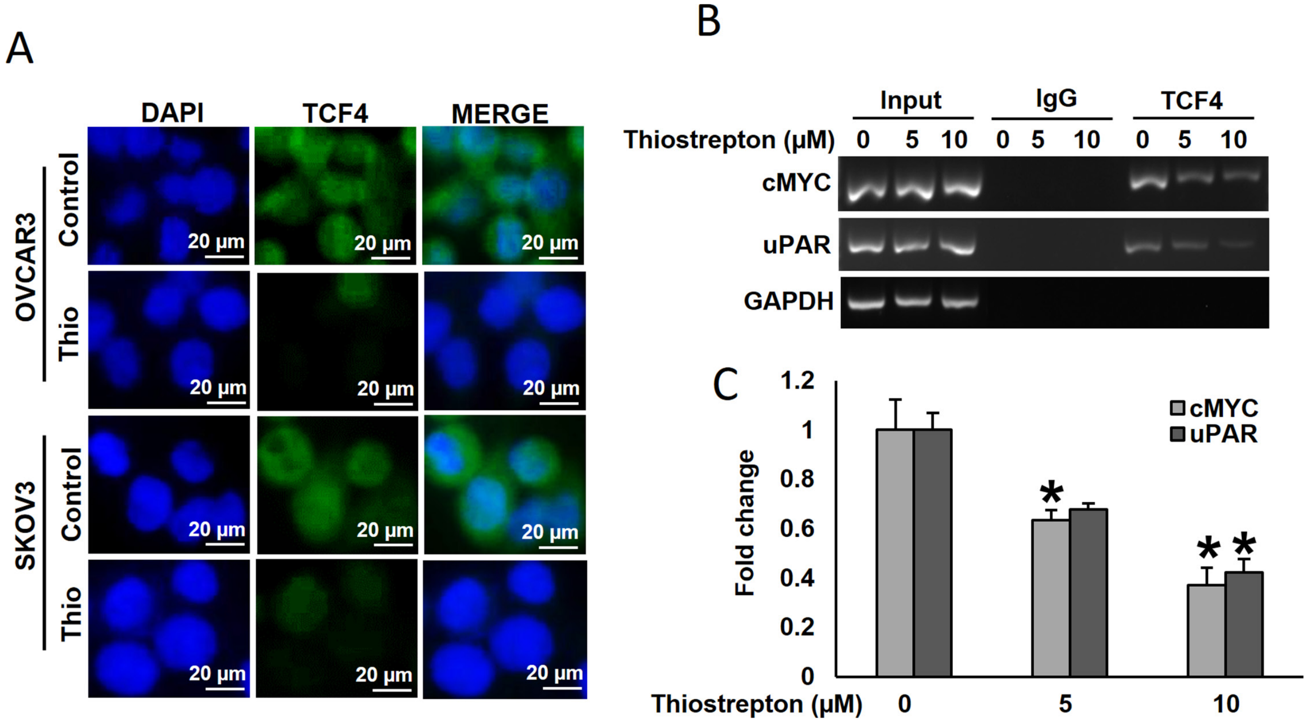

Supplementary Figure 1: Thiostrepton inhibits TCF4 expression and binding of TCF4 to cMYC and uPAR promoters. (**A**) Thiostrepton inhibits TCF4 expression as represented by fluorescence immunostaining in EOC cells. (**B**–**C**) Thiostrepton inhibits binding of TCF4 to cMYC and uPAR promoters. For the ChIP analysis, the TCF4 binding regions on cMYC and uPAR promoters were identified. OVCAR3 cells were treated with and without indicated doses of thiostrepton. After 48 hours, cells were fixed with formaldehyde and cross-linked. The chromatin was sheared and immunoprecipitated with anti-TCF4 antibody or control mouse IgG. The TCF4 binding to the cMYC and uPAR promoters was analyzed using specific primers. $^{*}p < 0.05$, statistically significant difference from control cells.

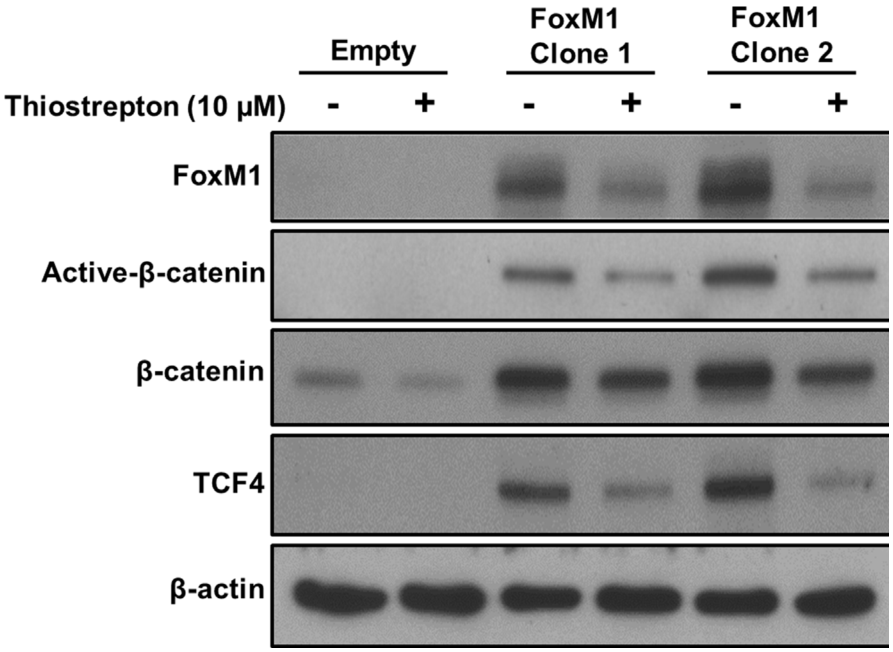

Supplementary Figure 2: Thiostrepton inhibits FoxM1 and its down-stream targets in FoxM1 overexpressing OVTOKO clones. FoxM1 overexpressing OVTOKO clones were treated with thiostrepton (10 μM) for 48 hours. After cell lysis, equal amounts of proteins were separated by SDS-PAGE, transferred to immobilon membrane, and immuno-blotted with antibodies against FoxM1, active-β-catenin, β-catenin, TCF4 and β-actin as indicated.

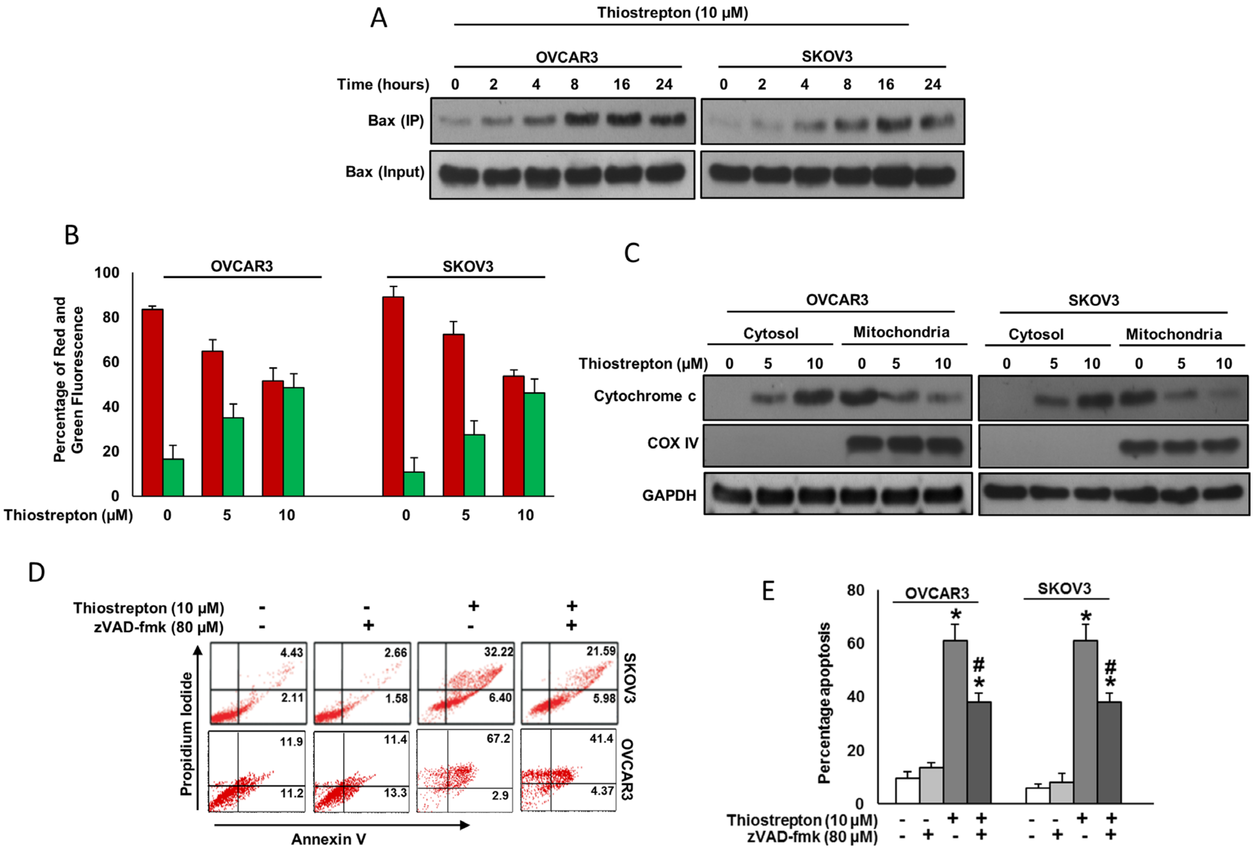

Supplementary Figure 3: Thiostrepton induced Bax activation and loss of mitochondrial potential in EOC cells. (**A**) Thiostrepton induced Bax activation in EOC cell lines.EOC cells were treated with thiostrepton (10 µM) for indicated time periods. Following treatment, cells were lysed in 1% CHAPS lysis buffer and subjected to immuno-precipitation with anti-Bax 6A7 monoclonal antibody and probed with specific polyclonal anti-Bax antibody (top band) for detection of conformationally changed Bax protein. In addition, the total cell lysates (bottom band) were applied directly to SDS–PAGE, transferred to immobilon membrane and immuno-blotted with specific anti-Bax polyclonal antibody. Following treatment, cells were lysed in 1% CHAPS lysis buffer and subjected to immunoprecipitation with either anti- BAX antibody or non-specific IgG as indicated for detection of confirmationally changed Bax protein. Proteins were separated on SDS-PAGE and immunoblotted with Bax rabbit polyclonal antibody. (**B**) Loss of mitochondrial membrane potential by thiostrepton treatment in EOC cells. EOC cells were treated with indicated doses of thiostrepton for 48 hours. Live cells with intact mitochondrial membrane potential (red bar) and dead cells with lost mitochondrial potential (green bar) was measured by JCI staining and analyzed by flow cytometry as described in materials and methods. The experiments were repeated at-least three times, and representative data are presented. (**C**) Thiostrepton induced release of cytochrome c. EOC cells were treated with indicated doses of thiostrepton for 48 hours. Mitochondrial and cytoplasmic fractions were isolated as described in Materials and methods. Cell extracts were separated on SDS-PAGE, transferred to PVDF membrane and immunoblotted with antibodies against cytochrome c and GAPDH. (**D–E**) Thiostrepton induced apoptosis is caspase dependent. EOC cells were pre-treated with universal caspase inhibitor, zVAD-fmk (80 µM) for 3 hours and subsequently treated with 10 µM thiostrepton for 48 hours and apoptosis was measured by flow cytometry after staining with Fluorescein annexin v/PI dual staining. Data presented in the bar graphs are the mean ± SD of three independent experiments. *indicates a statistically significant difference compared with untreated control with $p < 0.05$.# indicates a statistically significant difference compared with thiostrepton treated alone with $p < 0.05$.

Supplementary Table 1: Association of clinico-pathological characteristics with co-expression of Fox-M1 & β-catenin in patients with epithelial ovarian cancer

	Total		High Fox-M1 + β-catenin		Low Fox-M1 + β-catenin		*p* value
	N	%	*N*	%	*N*	%	
Total Number of Cases	252		32	12.7	220	87.3	
Age							
≤ 50 years	115	45.6	11	9.6	104	90.4	0.1670
>50 years	137	54.4	21	15.3	116	84.7	
Histopathology							
High grade Serous	134	53.2	117	87.3	17	12.7	0.0679
Low grade Serous	52	20.6	48	92.3	4	7.7	
Mucinous	26	10.3	3	11.5	23	88.5	
Endometriod	27	10.7	8	29.6	19	70.4	
Clear cell	5	2.0	0	0.0	5	100.0	
Undifferentiated	8	3.2	0	0.0	8	100.0	
FIGO Grade							
Well differentiated	47	18.7	4	8.5	43	91.5	0.1442
Moderately Differemtiated	109	43.2	19	17.4	90	82.6	
Poorly Differentiated	96	38.1	9	9.4	87	90.6	
Tumour Stage							
Stage I–II	45	18.6	2	4.4	43	95.6	0.0389
Stage III–IV	197	81.4	29	14.7	168	85.3	

Contingency table analysis and χ^2 tests were used to study the relationship between clinicopathological variables and protein expression.
Survival curves were generated using the Kaplan-Meier method, with significance evaluated using the Mantel-Cox log-rank test.
The limit of significance for all analyses was defined as a *p*-value of 0.05.

Supplementary Table 2: Association of clinico-pathological characteristics with FoxM1 over-expression in patients with high grade serous epithelial ovarian cancer

	Total		High FoxM1		Low FoxM1		*p* value
	N	%	*N*	%	*N*	%	
Total Number of Cases	138		97	70.3	41	29.7	
Age							
≤50 years	56	40.6	38	67.9	18	32.1	0.6061
>50 years	82	59.4	59	71.9	23	28.1	
Tumour Stage							
Stage I	9	6.6	8	88.9	1	11.1	0.4381
Stage II	5	3.7	4	80.0	1	20.0	
Stage III	91	66.9	61	67.0	30	33.0	
Stage IV	31	22.8	23	74.2	8	25.8	
Ki-67							
Above 50	82	61.6	66	80.5	16	19.5	0.0072
Below = 50	51	38.4	30	58.8	21	41.2	
β-catenin							
Above 0	18	13.4	17	94.4	1	5.6	0.0089
Below = 0	116	86.6	79	68.1	37	31.9	
TCF4							
Above 90	121	89.6	88	72.7	33	27.3	0.2385
Below = 90	14	10.4	8	57.1	6	42.9	
VEGF							
Above 140	21	15.4	14	66.7	7	33.3	0.6122
Below = 140	115	84.6	83	72.2	32	27.8	
MMP-2							
Above 80	109	80.7	80	73.4	29	26.6	0.2403
Below = 80	26	19.3	16	61.5	10	38.5	
MMP-9							
Above 80	101	80.2	76	75.2	25	24.8	0.0268
Below = 80	25	19.8	13	52.0	12	48.0	
Cyclin D1							
Above 15	23	16.8	18	78.3	5	21.7	0.3773
Below=15	114	83.2	79	69.3	35	30.7	
u-PAR							
Above 50	102	76.1	75	73.5	27	26.5	0.2383
Below = 50	32	23.9	20	62.5	12	37.5	

Contingency table analysis and χ^2 tests were used to study the relationship between clinicopathological variables and protein expression.

Survival curves were generated using the Kaplan-Meier method, with significance evaluated using.the Mantel-Cox log-rank test.

The limit of significance for all analyses was defined as a *p*-value of 0.05

Supplementary Table 3: Association of clinico-pathological characteristics with FoxM1 over-expression in patients with low grade serous epithelial ovarian cancer

	Total		High FoxM1		Low FoxM1		*p* value
	N	%	*N*	%	*N*	%	
Total Number of Cases	55		26	47.3	29	52.7	
Age							
≤50 years	27	49.1	10	37.0	17	63.0	0.1340
>50 years	28	50.9	16	57.1	12	42.9	
Tumour Stage							
Stage I	3	6.0	1	33.3	2	66.7	0.3037
Stage II	2	4.0	2	100.0	0	0.0	
Stage III	40	80.0	18	45.0	22	55.0	
Stage IV	5	10.0	3	60.0	2	40.0	
Ki-67							
Above 50	19	35.2	9	47.4	10	52.6	0.9327
Below = 50	35	64.8	17	48.6	18	51.4	
β-catenin							
Above 0	9	17.3	4	44.4	5	55.6	0.8102
Below = 0	43	82.7	21	48.8	22	51.2	
TCF4							
Above 90	41	75.9	21	51.2	20	48.8	0.1921
Below = 90	13	24.1	4	30.8	9	69.2	
VEGF							
Above 140	10	18.9	7	70.0	3	30.0	0.1372
Below = 140	43	81.1	19	44.2	24	55.8	
MMP-2							
Above 80	38	71.7	20	52.6	18	47.4	0.0817
Below = 80	15	28.3	4	26.7	11	73.3	
MMP-9							
Above 80	40	83.3	20	50.0	20	50.0	0.5159
Below = 80	8	16.7	3	37.5	5	62.5	
Cyclin D1							
Above 15	15	27.8	11	73.3	4	26.7	0.0199
Below = 15	39	72.2	15	38.5	24	61.5	
u-PAR							
Above 50	38	73.1	20	52.6	18	47.4	0.1172
Below = 50	14	26.9	4	28.6	10	71.4	

Contingency table analysis and χ2 tests were used to study the relationship between clinicopathological variables and protein expression.
Survival curves were generated using the Kaplan-Meier method, with significance evaluated using the Mantel-Cox log-rank test.
The limit of significance for all analyses was defined as a *p*-value of 0.05.

Supplementary Table 4: Clinico-pathological variables for the epithelial ovarian cancer patient cohort (*n* = 276)

	***n* (%)**
**Age**	
Median	53.0
Range (IQR)^	42.0 – 63.0
**Histopathology**	
High grade Serous	143 (51.8)
Low grade Serous	61 (22.1)
Mucinous	28 (10.1)
Endometrioid	28 (10.1)
Clear cell	6 (2.3)
Undifferentiated	10 (3.6)
**Grade**	
Well differentiated	55 (19.9)
Moderately differentiated	121 (43.8)
Poorly differentiated	100 (36.3)
**Stage**	
I	34 (12.3)
II	14 (5.1)
III	167 (60.5)
IV	50 (18.1)
Unknown	11 (4.0)
**Progression free survival duration in months**	
Median	11.0
Range (IQR)^	5.0 – 23.0

Abbreviations - ^Inter quartile range.

Supplementary Table 5: Antibodies used for TMA IHC analysis

Antibody	Clone	Company	Source	Dilution*	Retrieval	Detection Kit
FoxM1	G-5	SCBT (Santa Cruz, CA)	Mouse	1:2000	pH 6	Envision +
Ki-67	MIB-1	Dako (Carpinteria, CA)	Mouse	1:500	pH 9	Envision +
β-catenin	14	BD Biosciences (San Jose, CA)	Mouse	1:500	pH 6	Envision +
TCF4	mono-EP2033Y	Abcam (Cambridge, UK)	Rabbit	1:100	pH 9	Envision +
MMP-2	42-5D11	Calbiochem (San Diego, CA)	Mouse	1:200	pH 6	Envision +
MMP-9	56-2A4	Calbiochem (San Diego, CA)	Mouse	1:2000	pH 6	Envision +
VEGF	polyclonal	Novus Biologicals (Littleton CO, USA)	Rabbit	1:2000	pH 6	Envision +
u-PAR	E3	SCBT (Santa Cruz, CA)	Mouse	1:50	pH 9	Envision +
Cyclin-D1	EP12	Dako (Carpinteria, CA)	Mouse	1:50	pH 9	Envision +

*Overnight incubation.
